# Effects of Two Competitive Soccer Matches on Landing Biomechanics in Female Division I Soccer Players

**DOI:** 10.3390/sports7110237

**Published:** 2019-11-14

**Authors:** Benjamin J. Snyder, Randolph E. Hutchison, Christopher J. Mills, Stephen J. Parsons

**Affiliations:** Department of Health Sciences, Furman University, Greenville, SC 29613, USA; randolph.hutchison@furman.edu (R.E.H.); chris.mills@furman.edu (C.J.M.); stephen.parsons@furman.edu (S.J.P.)

**Keywords:** fixture congestion, anterior cruciate ligament, football, ground reaction force, anterior tibial shear, kinetics, kinematics, sidestep cut

## Abstract

Fatigue has been proposed to increase the risk of knee injury. This study tracked countermovement jump, knee isometric strength, and kinetics and kinematics in 8 female soccer players (experimental group) during an anticipated sidestep maneuver before and after two matches played over a 43-h period. Time points were: Before and after match 1 (T0 and T1), 12 h after the first match (T2), and immediately after the second match (T3). A control group participated only in practice sessions. Isometric knee extension strength decreased by 14.8% at T2 (*p* = 0.003), but knee flexion was not affected until T3, declining by 12.6% (*p* = 0.018). During the sidestep maneuver, knee joint degrees of flexion at initial contact was increased by 17.1% at T3, but maximum knee and hip angle at initial contact were unchanged. Peak resultant ground reaction force (GRF) increased by 12.6% (*p* = 0.047) at T3 (3.03 xBW) from 2.69 xBW at T0, while posterior GRF was significantly higher than T0 at all three subsequent time points (T1 = 0.82 ± 0.23 xBW, T2 = 0.87 ± 0.22 xBW, T3 = 0.89 ± 0.22 xBW). Anterior tibial shear force increased significantly (*p* = 0.020) at T3 (1.24 ± 0.12 xBW) compared to T1 (1.15 ± 0.13 xBW), an 8.8% increase. Lateral tibial shear force was significantly higher at both T1 (0.95 ± 0.20 xBW) and T3 (1.15 ± 0.38 xBW) compared to T0 (0.67 ± 0.25 xBW). These findings suggest that participation in a soccer match has significant effects on both physical performance parameters and kinetics/kinematics during a sidestep cut, but these can be more pronounced after a second match with short rest.

## 1. Introduction

Injuries to the anterior cruciate ligament (ACL) continue to be all too common in sports, and cause both short-term and long-term cost and medical problems [1,2]. Women are significantly more likely than men to suffer ACL injury [3,4], and most of those are non-contact in nature [5,6]. There is higher risk of injury during change of direction maneuvers, and increased anterior tibial forces are thought to be associated with higher strain on the ACL [5].

Soccer players are at especially high risk for ACL injury [3], and the fatiguing nature of the sport adds an extra dimension, as fatigue has been hypothesized to play a role in increasing ACL injury risk [6,7]. Numerous studies have tested the effects of a single actual or simulated soccer match on markers of physical performance, including repeated sprint ability, vertical jump, and maximum voluntary leg strength, and almost all have found consistent effects of fatigue lasting as long as 48–72 h post-match [8,9]. However, several recent reviews point out that the kinematic and kinetic risk factors for non-contact ACL injury are not consistently present after a single bout of fatiguing exercise [10,11,12].

Multiple matches played with short rest periods between them, also known as fixture congestion, is an often-mentioned concern of coaches and players. A number of studies have investigated the effect of fixture congestion distance covered and sprint distance or intensity [13,14,15,16,17], number of sprints [15], and incidence of injuries [13,14,15] in comparison to matches with more rest in between. Most have found no group differences, although one highlighted a higher injury rate in the second game in a week [15], and another identified small decreases in total distance and small increases in low intensity running—in particular, 15-min epochs by players participating in matches with less than four days of rest [16]. The studies all approached the problem differently with regards to the number of participants, number of matches measured, and period of time covered, but almost all used some aspect of running performance as the dependent variable without considering changes in physical performance aspects like vertical jump or leg strength that may be affected for some period of time after the match has concluded.

To our knowledge, only one study to date [18] measured changes in physical performance parameters following two soccer matches, finding that concentric knee extension was decreased up to 48 h after the first game before recovering at 72 h, before decreasing and remaining lower at 48 h after the second match. Eccentric knee flexion strength did not recover before the second match, 72 h later, remaining lower for 48 h after the second match. Likewise, countermovement jump was lower throughout the length of the study. However, this study used only males and did not measure ACL injury risk factors.

Some athletic conferences in Division I women’s soccer use a unique match pattern where teams compete twice in a 43-hour period, presenting an opportunity to observe the effects of accumulated match fatigue with much less rest than is considered necessary for effective recovery. Given that a number of biomechanical factors, including ground reaction forces (GRFs) [19,20] and joint forces (especially anterior tibial shear force [5,21,22]), are related to increased risk for ACL injury, and since fatigue may result in changes to these factors, this study was designed to investigate the fatiguing effects of match play and changes in selected ACL injury risk factors after two soccer matches with a short rest period. Based on the research cited above, we hypothesized that physical performance characteristics would decline after both matches, but that biomechanical factors were more likely to be affected after the second match.

## 2. Materials and Methods

### 2.1. Participants

**Figure 1:**
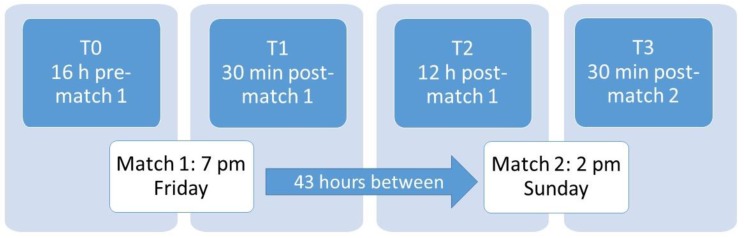
Project timeline.

Members of a women’s Division I soccer team were directly recruited before the season began and all players signed a consent form to participate. Because it was unclear which players would play the most minutes, and not all players could be tested due to time constraints, all 32 players were informed that they may be asked to participate depending on their playing time. Players with previous lower limb injury, specifically injury to the knee or ankle joint, and players who were injured during the course of the study were excluded. As each testing window approached, researchers consulted with the head coach to select the players most likely to receive the most game time, and these players were informed to prepare for the testing procedure that week. Players who exceeded 7.5 km traveled per game during both matches were put into the experimental group and classified as match play (MP) (*n* = 8, mean body weight (BW) = 63.5 ± 6.3, mean height = 168.3 ± 5.6 cm). Two weeks later, seven additional players who did not participate in games were classified as the control (CON) group (*n* = 7, mean BW = 61.0 ± 6.6 kg, mean height = 167.8 ± 6.6) and were tested at the same time points while only participating in practices. Because the testing window occurred after games, at least one of which ended at 9:00 pm, it was impossible to test both MP and CON groups in the same week. Muscle function was tested using counter movement jump (CMJ), Margaria–Kalamen (M–K) power, maximal voluntary isometric contraction (MVIC) of knee flexion (KF) and knee extension (KE), while kinetic/kinematic parameters were collected to assess risk for knee injury. The schedule for measurements is depicted in Figure 1. The study was reviewed and approved by the University’s Institutional Review Board following the principles of the Declaration of Helsinki prior to the initial recruitment of participants. 

### 2.2. Study Procedure

Match play in the conference regularly includes several instances of a Friday 7:00 pm/Sunday 2:00 pm series. To test the hypothesis, three tests of power/strength and kinematic/kinetic measurements were completed at four time points around the two matches: 16 h prior to the first match (T0), 30 min after the first match (T1), 12 h after the first match and 28 h before the second match (T2), and 30 min after the second match (total of 43 h after the completion of the first match, T3). These time points were chosen in consultation with the head coach to minimize disruption of match preparation. For T0, participants (including controls) reported to the lab immediately after a walk-through practice where the average distance traveled was less than 1.5 km for those who participated (3 players had rested) and completed the physical performance tests in the following order: CMJ, M–K power test, and MVIC. They then proceeded to the motion capture lab in the order that they completed the prior testing, and after changing into compression shorts and a sports bra, were fitted with retroreflective markers. For T2, participants began their testing immediately after a light pool workout designed to relieve muscle soreness. Thus, for each time point, participants were adequately warmed up to begin testing immediately.

### 2.3. Physical Performance Measurements

#### 2.3.1. Global Positioning System (GPS) Movement Data

A GPS unit (Viper pod, Statsports, Newry, Northern Ireland), mounted in a neoprene pocket affixed between the shoulder blades to a sports bra, reported location at 10 Hz and accelerometry at 100 Hz. Following matches and practice, data were downloaded to a laptop computer and analyzed with proprietary software (Statsports). Total distance, accelerations and decelerations exceeding 2× the force of gravity, and sprints exceeding 80% of the player’s previously tested max speed for greater than 1 second were collated and analyzed. Data for matches are found in Table 1.

#### 2.3.2. Countermovement Jump

Participants were told to jump as high as possible and displace the highest possible vane on a freestanding Vertec apparatus (Sports Imports, Columbus, OH, USA). Using a no-step countermovement jump, participants attempted three trials with a 30 second rest period between attempts and the highest jump was recorded in inches and later converted to centimeters (cm).

#### 2.3.3. Margaria–Kalamen Test

Following previously published procedures [23], participants used a 6 m run-up and climbed a set of 9 stairs with an individual height of 0.106 m each as quickly as possible. Timing mats (Lafayette Instruments, Layfayette, IN, USA) were placed on the third and ninth steps, and power was calculated as BW (kg) × 9.8 × total step height/time. Participants completed three satisfactory trials with a 30 second rest period between attempts and the highest power was used.

#### 2.3.4. Maximum Voluntary Isometric Strength

Due to time constraints and limited access to an isokinetic measurement device in close proximity to the soccer field, isometric measurements were used to measure muscle strength. Participants were seated on a chair with the seat height adjusted to keep their feet off the ground and the seat back adjusted to center the knee joint just off the edge of the seat. A Velcro cuff attached to a strain gauge (Takei Scientific Instruments, Yashiroda, Japan) was placed around their ankle of their dominant leg as close to the top of the shoe as possible and attached by a chain to the support apparatus of the chair. Leg dominance was established by asking the athlete which leg they are most comfortable using to pass and shoot, and only one participant was left-footed. For the knee extension maximum voluntary isometric contraction (MVIC), the length of the chain was adjusted to achieve a knee angle of 80° during the effort. For the knee flexion MVIC, the chain was attached to a vertical steel post and the length adjusted to achieve a knee angle of 120° during the effort. A Velcro strap around the waist held participants to the seat back, and a leather belt was placed around both legs just proximal to the knee to prevent the leg from lifting off the seat during the effort. For each MVIC, participants were told to gradually increase their force production during a 3-second countdown and then hold maximal effort for 2 seconds. Verbal encouragement was provided during each effort. Two repetitions were completed with 1 min of rest between each. If the two trials differed by more than 10%, a third repetition was completed and the two most consistent efforts were averaged and recorded in kilograms.

### 2.4. Biomechanical Measurements

A rigid body model was created to correlate the location of motion tracking markers with anatomical landmarks, and was scaled to match subject-specific measurements. International Socity of Biomechanics (ISB) recommendations were followed to create Cartesian coordinate systems for each segment, defined at the center of mass of each segment, as well as joint coordinate systems (JCS) based on anatomical landmarks with the first axis embedded in the proximal segment and chosen to represents flexion-extension, the “floating” axis chosen to represent ad-abduction, and the third axis embedded in the distal segment and chosen to represent axial rotation of that segment [24,25] The center of gravity of each segment, including the pelvis, was defined by using the geometric center of mass of the frustrum of a right circular cone. The length, proximal radius, and distal radius of each cone defined this shape based on the anatomical landmarks [26]. Joint position, velocity, acceleration, and internal moment were calculated from a six degrees of freedom retro reflective marker set placed on the subject’s lower extremity and pelvis. Seven segments were defined, including bilateral foot, shank, thigh, and single pelvis. The joint reaction forces (shear forces) were normalized to subject mass 35. A total of 17 markers were placed on the following landmarks on both limbs: Superior sacrum, anterior superior iliac spines, just proximal to the patella, femoral condyles, tibial tuberosities, lateral malleoli, second metatarsals, fifth metatarsals, and the calcaneus at the insertion of the Achilles tendons. These markers were used in conjunction with an 8-camera, 240 Hz motion capture system (ProReflex MCU 240, Qualisys, Gothenburg, Sweden) to capture three-dimensional (3D) motion associated with the 45 degree sidestep. In addition to the 3D motion, the peak ground reaction force was collected during the stance phase for each subject using a force plate (AMTI LG6-4-200, Advanced Mechanical Technology, Inc., Watertown, MA, USA) embedded in the ground, measuring at a sampling rate of 1920 Hz. Initial contact was defined by the onset of the GRF greater than 10 N and ending with toe-off. Timing gates (FarmTek, Inc., Wylie, Texas) were also used to ensure that participants were running and cutting on the force plate at a pace between 4–5 m/s [27]. Data were collected with Qualysis Track Manager (Gothenburg, Sweden). Kinematic data and inverse dynamic calculations were used to determine joint reaction forces (tibal shear force) in the anteroposterior and mediolateral planes using Visual 3D software (C-Motion Inc., Germantown, MD, USA). Signals were processed with a 20 Hz Butterworth low pass filter.

#### 2.4.1. Procedures

Before testing, participants were given a brief overview of the study protocol. Participants then had markers placed at major landmarks on the lower limbs of the body. Each subject was then familiarized with the sidestep maneuver on the force plate (AMTI LG6-4-200, Advanced Mechanical Technology, Inc., Watertown, MA, USA). Each subject was instructed to approach the force plate at the set velocity (4–5 m/s), plant with their dominant foot, and run away from the force plate to the contralateral side at a 45-degree angle along a clearly marked pathway. Several practice trials were performed to ensure correct pace and correct execution of the maneuver (running directly at the force plate, striking the center of the force plate, no stutter step) when executing the sidestep maneuver. Participants then performed three acceptable trials each for the sidestep on the force plate, with a 1 min rest between trials. Trials were repeated if the correct speed was not achieved or participants did not execute the maneuver correctly. Data from these three trials were averaged.

#### 2.4.2. Statistical Analysis

All statistics were carried out with the IBM SPSS software package (IBM SPSS, Version 25, Armonk, NY, USA). A two-way mixed model repeated measures analysis of variance was used to test for differences physical performance parameters and kinetic and kinematic changes, using a group x time x group*time model. A significant group*time interaction (*p* ≤ 0.05) indicated group differences, whereas main effects were examined post-hoc with univariate pairwise comparisons, with *p*-value of 0.05 as the cutoff for significant differences. Paired *t*-tests were used to compare GPS-derived values between game 1 and 2, with a *p*-value of 0.05 as the cutoff for significant differences. All values were expressed ± the standard deviation (SD). Effect sizes (*d*) were calculated and indexed as small (*d* < 0.2–0.5), medium *d* ≥ 0.5, but <0.8), and large (*d* ≥ 0.8) [28], and calculated as follows with M = sample mean and SD = standard deviation:

d = (M_2_ − M_1_)/SD_pooled_

## 3. Results

### 3.1. Strength and Power

**Figure 2:**
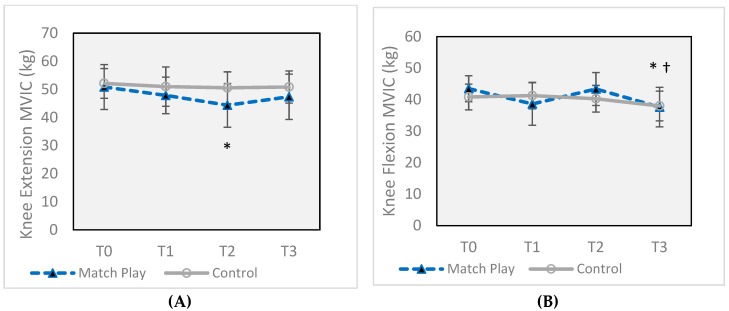
(**A**) Maximal voluntary isometric knee extension in kilograms before and after two soccer matches. (**B**) Maximal voluntary isometric knee flexion in kilograms before and after two soccer matches. Error bars indicate ± standard deviation (SD). * Significant difference from Match Play T0 (*p* ≤ 0.05). † Significant difference from Match Play T2 (*p* ≤ 0.05). T0 = 16 h prior to the first match, T1 = 30 min after the first match, T2 = 12 h after the first match, and T3 = 30 min after the second match (total of 43 h after the completion of the first match, T3).

**Figure 3:**
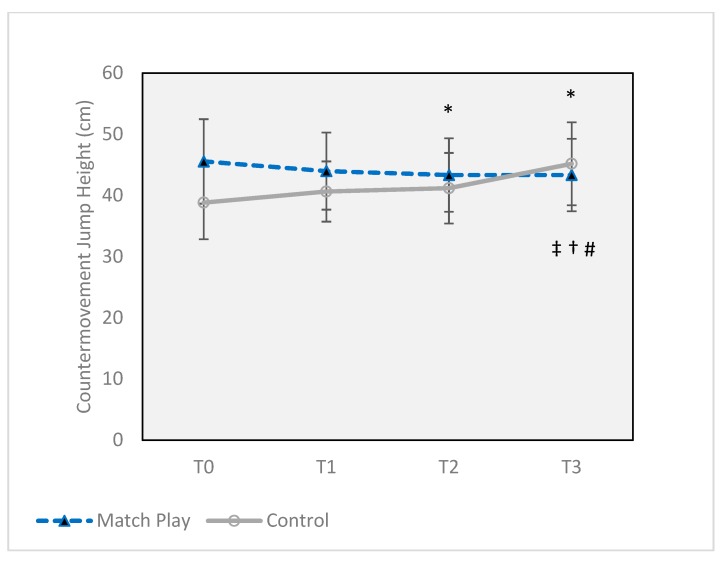
Countermovement jump height in centimeters before and after two soccer matches. Error bars indicate ± SD. * Significant difference from Match Play T0 (*p* ≤ 0.05). ‡ Significant difference from Control T0 (*p* ≤ 0.05). † Significant difference from Control T1 (*p* ≤ 0.05). # Significant difference from Control T2 (*p* ≤ 0.05). T0 = 16 h prior to the first match, T1 = 30 min after the first match, T2 = 12 h after the first match, and T3 = 30 min after the second match (total of 43 h after the completion of the first match, T3).

In the MP group, KE (Figure 2A) was significantly decreased at T2 (44.4 ± 7.8 kg, *p* = 0.003) compared to T0 (50.9 ± 8.0 kg) (*p* = 0.003, *d* = 0.82), a 14.8% decline, but was returned to baseline at T3 (47.4 ± 8.1 kg). KF (Figure 2B) was decreased from baseline values (43.5 ± 4.1 kg) by 12.6% at T3 (37.6 ± 6.3 kg, *p* = 0.018) with a large effect size (*d* = 1.1), but was unchanged at previous time points. There were no CON group differences in KF or KE MVIC. CMJ (Figure 3) increased at each successive time point in the CON group, with an overall 16% increase realized from T0 (38.8 ± 6.0 cm) to T3 (45.5 ± 6.8 cm) (*p* = 0.010, *d* = 0.93). In the MP group, CMJ was decreased at both T2 and T3 (43.3 ± 5.9 cm) by 4.9% (*p* = 0.021) compared to T0 (45.6 ± 6.9 cm), but with a small effect size (*d* = 0.34). There were no changes to M–K power for either group.

### 3.2. Kinematic and Kinetic Parameters

**Figure 4:**
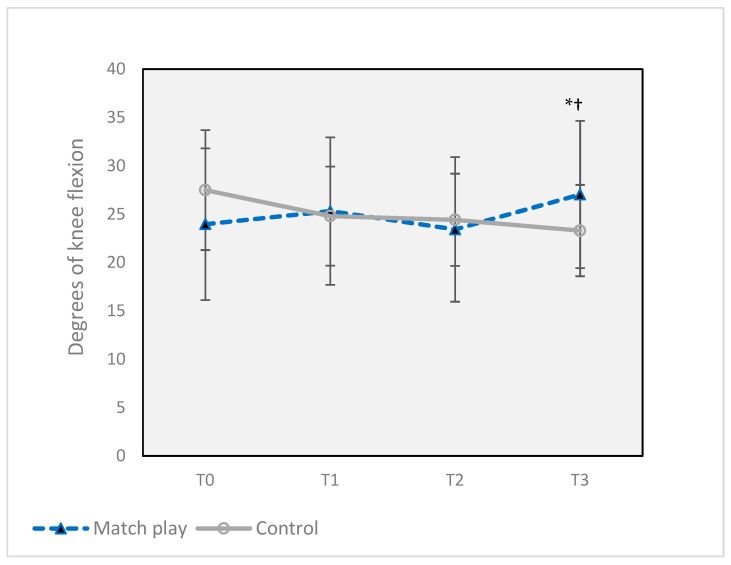
Changes in knee flexion angle at initial contact during a sidestep maneuver before and after two soccer matches. Error bars indicate ± SD. * Significant difference from Match Play T0 (*p* ≤ 0.05). † Significant difference from Match Play T2 (*p* ≤ 0.05). T0 = 16 h prior to the first match, T1 = 30 min after the first match, T2 = 12 h after the first match, and T3 = 30 min after the second match (total of 43 h after the completion of the first match, T3).

There were no changes in any kinetic or kinematic parameters in the control group at any time point. Knee joint degrees of flexion at initial contact (Figure 4) was increased from 24.0 ± 7.8° at T0 to 27.1 ± 7.6° at T3, a 17.1% increase (*p* = 0.010) with a small effect size (*d* = 0.48), but max knee angle and hip angle at initial contact were unchanged for the MP group.

**Figure 5:**
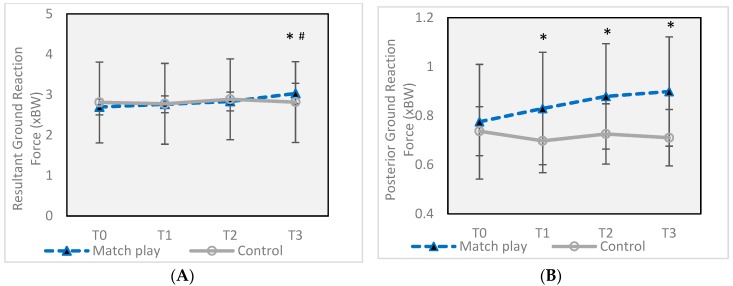
(**A**) Changes in peak resultant ground reaction force during a sidestep maneuver before and after two soccer matches. (**B**) Changes in peak posterior ground reaction force during a sidestep maneuver before and after two soccer matches. Error bars indicate ± SD. * Significant difference from Match Play T0 (*p* ≤ 0.05). # Significant difference from Match Play T1 (*p* ≤ 0.05). T0 = 16 h prior to the first match, T1 = 30 min after the first match, T2 = 12 h after the first match, and T3 = 30 min after the second match (total of 43 h after the completion of the first match, T3).

Peak resultant ground reaction force (GRF) (Figure 5A) increased significantly from 2.69 ± 0.20 Newtons/Newtons body weight (xBW) at T0 to 3.03 ± 0.25 at T3 (*d* = 1.49), a 12.6% increase (*p* = 0.047), while posterior GRF (Figure 5B) was significantly higher than T0 (0.77 ± 0.23 xBW) at all three subsequent time points (T1 = 0.82 ± 0.23 xBW (*p* = 0.046), T2 = 0.87 ± 0.22 xBW (*p* = 0.013), T3 = 0.89 ± 0.22 xBW (*p* = 0.020)). Only the difference at T3 registered a moderate or higher effect size, with *d* = 0.54, representing a 15.9% increase. There was no difference in mediolateral GRF at any time point in the MP group.

**Figure 6:**
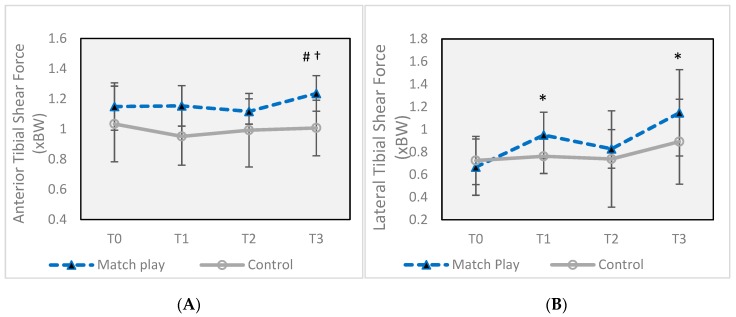
(**A**) Changes in peak anterior tibial shear force during a sidestep maneuver before and after two soccer matches. (**B**) Changes in peak lateral tibial shear force during a sidestep maneuver before and after two soccer matches. Error bars indicate ± SD. * Significant difference from Match Play T0 (*p* ≤ 0.05). # Significant difference from Match Play T1 (*p* ≤ 0.05). † Significant difference from Match Play T2 (*p* ≤ 0.05). T0 = 16 h prior to the first match, T1 = 30 min after the first match, T2 = 12 h after the first match, and T3 = 30 min after the second match (total of 43 h after the completion of the first match, T3).

Anterior tibial shear force (Figure 6A) increased significantly (*p* = 0.020) at T3 (1.24 ± 0.12 xBW) compared to T1 (1.15 ± 0.13 xBW), an 8.8% increase with a moderate effect size of 0.71, but was not significantly different from T0 (*p* = 0.054). Lateral tibial shear force (Figure 6B) was significantly higher at both T1 (0.95 ± 0.20 xBW, *p* = 0.016) and T3 (1.15 ± 0.38 xBW, *p* = 0.020) compared to T0 (0.67 ± 0.25 xBW) with large effect sizes of 1.24 and 1.49, respectively. The increase at T1 represented a 42% increase over T0, while the change at T3 was an increase of 70%.

For the MP group, there were no significant differences between minutes played, total distance, number of accelerations or decelerations, or number of sprints performed in the first match compared to the second match (Table 1).

## 4. Discussion

This study sought to examine the effects of two consecutive soccer matches, with 43 h of rest in between, on physical performance characteristics and biomechanical parameters during a sidestep cutting maneuver. The results of the study show that while a single soccer match can change some biomechanical factors related to knee injury risk, the risk is significantly enhanced by participation in a second match with less than 48 h of rest for those athletes playing the greatest number of minutes. Following the first match, posterior GRF (Figure 5B) was increased by 7.1% (small effect size) and lateral tibial shear force (Figure 6B) was increased by 42% (large effect). However, following the second match, peak resultant GRF (Figure 5A) and anterior tibial shear force (Figure 6A) were also increased, in addition to further increases in posterior GRF and lateral tibial shear force, all with large effect sizes, except for posterior GRF (moderate effect). Values for both posterior and resultant GRF were very similar to values from previous research on female soccer players performing a sidestep cut, which ranged from 2.6–2.8 xBW for vertical GRF and 0.7–0.8 xBW for posterior GRF [29].

The changes to anterior tibial shear force were especially interesting, since the ACL is primarily responsible for resisting anterior tibial translation [30], and a number of studies have demonstrated the significant contribution of anterior tibial shear to ACL injury [5,21,22], especially in combination with axial compression and knee abduction [31]. The increased posterior GRF seen in our study likely contributed to the increased shear force, while increased resultant GRF likely increased tibial compression forces. Our study is the first to demonstrate the effects of fixture congestion on anterior tibial shear. Physical performance parameters were also affected. Knee extension MVIC (KE) was not affected immediately after the first match (T1), but was decreased 12 h later (T2) by 14.8%, while the change in KF only reached significance after the second match (T3), decreasing by 12.6%. These results are similar to those from previous research [18,32]. CMJ, an indicator of power production (Figure 3), was also unaffected at T1, but was decreased by 4.9% at both T2 and T3 (*p* ≤ 0.05). All but CMJ had large effect sizes.

This study is unique in several ways. First, although studies examining the effect of fatigue on ACL injury risk factors have used a variety of exercise methods, very few have used an actual soccer match. While there are disadvantages to using actual soccer matches instead of lab-based fatigue protocols (including controlling intensity of exercise and timeline of measurements), ACL injuries take place on the field, and therefore, measurements taken from match play will provide more directly applicable information. Overall, as an excellent review of studies between 2000–2016 indicates [10], there is little consensus on the effects of fatiguing exercise on ACL injury risk. Of the 37 studies cited by the review, only one utilized a 45-min soccer match as the fatiguing protocol, and none used a full match. Out of five studies published since 2000 using a planned sidestep cutting maneuver following a general fatigue protocol, only one [33] showed a change in knee flexion angle (decreased), while the others did not report significant changes [29,34,35,36]. Of the two studies reporting landing forces, none showed any changes. A study using a stop-jump task after fatiguing activities showed increased anterior tibial shear force [6], but this does not appear to be a consistent effect of fatigue [37]. Our novel finding of increased resultant and posterior GRF, as well as increased anterior tibial shear vis-a-vis other studies using sidestep cutting, may be due to the unique physical challenge of actual soccer matches as a fatigue protocol.

Second, although numerous studies have tracked physical performance parameters over time following a competitive soccer match, none included more than one match. The effect of “fixture congestion”, playing more than one match or a run of matches with less than normal rest, has been examined before, but most monitored changes to in-match parameters such as distance covered and sprint distance or intensity [13,14,15,16,17], number of sprints [15], and incidence of injuries [13,14,15]. The current study is the first to observe the effect of fixture congestion on physical performance parameters such as muscle strength or CMJ.

Lastly, although the effects of fatigue on ACL injury risk factors have been extensively investigated, the current study is the first to observe changes after a second match with less than normal rest. In doing so, the intent was to study chronic effects of intense exercise, therefore observing the cumulative and well-documented effects of muscle damage [8], as opposed to acute central or peripheral fatigue, on biomechanical indicators of risk.

Physical performance characteristics following a single bout of soccer-related activities have been examined extensively, and many of these studies used actual match play as the independent variable. Most have indicated a loss of leg strength ranging from 7–17% for knee extension, and 7–36% for knee flexion after a single match or simulated match [9]. A meta-analysis by Silva et al. [8] found that eccentric hamstring strength is decreased for 3 times as long as concentric quadricep strength, highlighting the different recovery period between the two muscle groups.

Few studies have measured the effects of multiple matches on performance parameters. One study tracked physical performance before and after two staged soccer matches separated by three days, and found different recovery kinetics for knee flexion vs. knee extension strength [18]. Their data showed that in the dominant limb, concentric knee strength was decreased by a maximum of 7.9% 24 h after the first match, and 9.4% 24 h after the second match, returning to baseline by 72 h. Following the second match, concentric knee strength had not returned to baseline values by 72 h. Eccentric knee strength decreased by 16.8% at 24 h and remained significantly below baseline through 72 h after the second match.

The results of the current study are not directly comparable due to a different timeline, the use of isometric strength measurements in the current study, and a subject pool consisting of all males, but also revealed different timelines for KE vs. KF (Figure 2). In our study, isometric knee extension strength declined by 14.8% after 12 h but recovered more quickly, exhibiting baseline levels at 45 h after the first match, despite a second match at 43 h. This makes it likely that KE had recovered fully between the measurement at 12 h and the second match and was not significantly affected by the second match (mean values at T3 were lower than baseline, at 47.4 kg vs. 52.1 kg, but not significantly so). Mean isometric KF was 11% lower after the first match, but this was not a significant change, unlike previously cited studies [18,32]. After the second match, it was nearly 13% lower (*p* = 0.18).

Another similar study [32] observed changes in knee strength after a soccer match (staged exclusively for the study), followed by two moderate to high practice sessions on day 1 and day 2 after the match. The all-male participants in the exercise group exhibited decreases in isometric, concentric, and eccentric strength that peaked at 12 h and failed to return completely to baseline by 60 h post-match. A control group that only participated in the practices also experienced a decline at 60 h, hinting at possible effects of the second practice session. Concentric and eccentric KF declined more than KE, an indicator of the eccentrically-dominant nature of knee flexor activity in soccer. The authors concluded that strength deficits can persist for a longer period than previously indicated when athletes continue to participate in training activities [32].

Collectively, these data show that studying the cumulative effects of multiple exercise bouts, whether lab- or field-based, may further elucidate the understanding of the effects of intense exercise on injury risk. It is especially important to note that non-synchronized KF and KE strength changes, confirmed by the current study and previous studies, have the potential to increase risk of ACL injury in a second match, given the effect of imbalance of flexor and extensor strength on knee mechanics, at least when the knee is somewhat flexed [38].

In our study, although there were no changes in hip or ankle angles due to match play, there was a 12.8% increase from baseline in knee flexion at T3. Based on existing literature, increased knee flexion during landing is desirable, since it can improve the mechanical advantage of the knee flexors, aiding the ACL in resisting potential anterior tibial shear [39]. However, in the current study, the decrease in KF strength at T3, combined with a recovery of KE strength to baseline levels and the small effect size (0.398) of the knee angle change, make a potential protective effect less likely.

Vertical jump is also affected by match play, but not consistently. From a selection of six recent studies observing effects of soccer match play on countermovement jump, two reported no changes, and four others reported decreases ranging from 4.4–12% within 12 h post-match [9,40]. Our result of no change immediately after one match but a decrease after 12 h is thus consistent with some studies; however, our finding of a decrease after a second match is novel. Also, the significant group interaction (*p* = 0.0001) when comparing MP to CON, with CON increasing their performance at T3 by 16% (*p* = 0.010), reveals there may have been a learning effect with a repeated CMJ test that may have mitigated the decrease in performance in MP participants.

### Limitations

The choice to use actual match play as the treatment, rather than simulated matches or lab-based exercise protocols, was based on evidence that actual match play is more physically demanding, with longer lasting physiological effects [8]. Additionally, the results derived from this study are highly generalizable to athletic populations, and to our knowledge, there are no existing data on biomechanical effects of actual match play. There are, however, several limitations introduced by this protocol. For example, the amount and intensity of exercise cannot be controlled by the investigators, potentially introducing a large difference in the effects of the match on different players. We sought to address this by limiting the subject pool to athletes that ran more than 7.5 km per game and played more than 60 min in both games. Likewise, other indices of workload, such as the number of accelerations, decelerations, and sprints above an individualized threshold, were tracked by GPS technology and were confirmed to be statistically identical across both matches. Therefore, despite the presence of interindividual differences in match workload, both matches represented the same challenge to the participants on average. These restrictions, however, had the effect of limiting the subject pool. Another limitation to the study is the lack of flexibility of the scheduling of data collection. For instance, an additional measurement of all factors a few hours before the match would have been desirable, but would have disrupted the pre-match preparations, and a follow-up at 72 h could not be scheduled for all athletes. Likewise, the use of isokinetic measures of leg strength would have provided interesting data, but the location of the equipment was not conducive to collecting the data without more serious disruption to the players’ post-game routine.

## 5. Conclusions

Female soccer players show some biomechanical and performance alterations after a single 90-min competitive match, including decreased knee extension strength, decreased countermovement jump, increased posterior GRF, and increased lateral tibial shear force. However, after a second match played within 43 h, additional effects are present, including increased knee flexion, decreased knee flexion strength, increased peak resultant GRF, and increased anterior tibial shear force, that were not apparent after the first match. Given the importance of anterior tibial shear force as a mechanism of ACL injury, the finding of increased anterior tibial shear combined with a recovery of knee extension strength indicates the potential for additional risk of knee injuries to players when there is insufficient recovery from match play.

## Figures and Tables

**Table 1:** GPS- and accelerometer-derived match activity. Values are given ± SD.

	Match 1	Match 2	Range (Both Matches)
**Minutes played**	77.5 ± 8.9	75 ± 12.2	63–90
**Distance traveled (km)**	8.64 ± 0.51	8.36 ± 0.54	7.5–9.8
**Decelerations**	42.3 ± 9.9	42.6 ± 4.3	28–62
**Accelerations**	32.1 ± 9.6	28.8 ± 7.2	18–50
**Sprints**	11.4 ± 9.5	9.1 ± 7.1	1–29
